# Phonon mode potential and its contribution to anharmonism

**DOI:** 10.1038/s41598-020-76454-y

**Published:** 2020-11-13

**Authors:** Paweł T. Jochym, Jan Łażewski, Wojciech Szuszkiewicz

**Affiliations:** 1 Institute of Nuclear Physics, Polish Academy of Sciences, Radzikowskiego 152, 31-342 Cracow, Poland; 2 Institute of Physics, College of Natural Sciences, University of Rzeszów, Pigonia 1, 35-310 Rzeszow, Poland; 3 Institute of Physics, Polish Academy of Sciences, Lotników 32/46, 02-668 Warsaw, Poland

**Keywords:** Semiconductors, Structure of solids and liquids

## Abstract

We present systematic ab-initio study on the phonon mode potential as a source of anharmonicity in the crystal. As an example, the transverse optical (TO) mode potential in PbTe has been fitted to density-functional-theory calculated energies of phonons excited with different amplitudes of mode displacements. The corresponding equation of motion has been analytically and numerically solved in 1D and 2D space, respectively. The solution is used for constructing the ensemble of 10,000 systems with potential and kinetic energies selected according to the thermal equilibrium distributions. The velocity auto-correlation function derived from the computed trajectories is then used to calculate the profile of the phonon spectrum for the TO an LA modes at three temperatures of 100, 300, and 600 K. This technique allows for determination of the contribution of non-quadratic potential of the phonon mode to the anharmonicity in the crystal and its effect on the phonon spectrum.

## Introduction

Anharmonicity in crystals is often understood as any deviation from the harmonic behaviour, regardless of its origin. The root cause of arising discrepancy can be generally identified with a non-quadratic shape of the vibrational mode potential (e.g. due to geometry of the bonding) or with some additional interactions between lattice vibration modes and other degrees of freedom in the crystal (e.g. other modes, magnons, electronic excitations). The classical harmonic theory^1^, while very successful, has its limitations. Namely, it is fundamentally unable to properly describe important phenomena rooted in phonon-phonon interactions (e.g. thermal equilibrium, thermal expansion or thermal conductivity, phase transitions and many others). In some cases, where the anharmonicity can be encapsulated into small corrections to the harmonic model, the description can be successfully extended to include some of the mentioned phenomena (e.g. thermal expansion^2^). Even phenomena connected with strong anharmonicity (e.g. phase transition) can be modelled, to some extent, using this approach^3–5^. However, such extended models are usually not general and have limited applicability to cases, where the anharmonic behaviour is strongly pronounced. In general, the harmonic model is applicable only when normal modes in the crystal are independent or weakly interacting with other degrees of freedom. Whereas in cases where normal modes are strongly interacting and/or exhibit significantly non-quadratic potential, the use of harmonic approximation, even extended with some corrections, becomes questionable.

These more difficult cases have been the subject of extensive research^6–17^ which has led to the development of a number of methods designed to deal with strong anharmonicity—e.g. with anharmonicity presumably present in the transverse optical (TO) mode of vibrations of the PbTe crystal^18^. These methods range from some form of corrections or additions to the harmonic model^19–26^ to building full anharmonic model for the crystal^27–32^ using either ab-initio (Density Functional Theory—DFT) based molecular dynamics as a source of the data for the model^31,33,34^ or some form of alternative approach^7,19,22,24,26^. Works mentioned above try to explain anomalous features of the PbTe spectrum by various aspects of anharmonicity in the PbTe crystal. However, they do not address explicitly the issue of supposed large anharmonicity of the TO mode itself^18^ and its influence on the whole phonon spectrum of the PbTe crystal. Our work aims to fill this gap in understanding of phonon properties of this material and possibly elucidate the role of mode potential in anharmonic lattice dynamics of other crystals.

Potential energy of the crystal can be described as a Taylor’s expansion with respect to the atomic displacement (${\mathbf {u}}$) around the equilibrium configuration (i.e. $u_i^\alpha = 0$, where *i* numbers atoms in the lattice and contains together indexes inside the primitive unit cell and lattice indexes of the unit cell in the crystal, while Greek letters $\alpha ,\beta \ldots$ number *x*, *y*, *z* directions). This formula can be expressed as a sum of consecutive orders of approximation^35^:

1 $$\begin{aligned} V =&V_0 + \sum \limits _{i,\alpha } \Phi _{1i}^{\ \alpha } u_i^\alpha + \frac{1}{2!}\sum \limits _{\begin{array}{c} ij\\ \alpha \beta \end{array}} \Phi _{2ij}^{\ \alpha \beta } u_i^\alpha u_j^\beta + \\&\quad \frac{1}{3!}\sum \limits _{\begin{array}{c} ijk\\ \alpha \beta \gamma \end{array}} \Phi _{3ijk}^{\ \alpha \beta \gamma } u_i^\alpha u_j^\beta u_k^\gamma + \ldots \nonumber \end{aligned}$$

where $n\text {th}$ rank tensors $\Phi _{nijk\ldots }^{\ \alpha \beta \gamma \ldots }$ are $n\text {th}$ derivatives of potential energy with respect to displacements $u_i^\alpha , u_j^\beta \ldots$, respectively. In harmonic approximation^1^, only the quadratic term is taken into account, which is equivalent to the small displacements approximation in the classical theory of oscillators. It is clear that limiting the expansion to quadratic terms, greatly simplifies the model and reduces number of free parameters. A further consequence of this assumption is linear independence of phonon modes, described by polarization vectors which form an orthonormal basis set.

It may be considered surprising that using harmonic models with non-interacting degrees of freedom, which when excited cannot reach thermal equilibrium, one can obtain quite a good agreement of calculated dynamical and thermodynamic properties with experimental data^36–38^. On the other hand, considering higher order terms in Eq. 1 allows for inclusion of multi-phonon interactions^20,28,29^ and introduces finite lifetime of the atomic vibrations manifesting in the broadening of phonon lines. In several papers published in recent years^18,39^ it is claimed that the strong, even gigantic, anharmonicity of the PbTe TO phonon mode near the zone center^18^ or strong anharmonic components in the inter-atomic potential^39^ lead to anomalous behavior of the transverse optical mode near the Brillouin zone center. Therefore, our main motivation to undertake present study was clarification to what extent anharmonism of the system, especially the shape and broadening of the phonon peaks, can be associated with characteristic features of the mode potential of this particular vibration. In other words, we will try to isolate the part of anharmonism originating from non-quadratic energy profile of the single phonon mode. We are building our case on few important assumptions. We need to assume that the anharmonicity in the system is small enough for the concept of isolated normal modes to be still valid. Furthermore, the mode potential should not depend strongly on other modes in the crystal (i.e. the normal modes are mostly independent). The last assumption is that the mode potential energy surface derived from DFT captures all essential processes determining vibrations of the lattice. It is clear that some effects are beyond this approximation—e.g. dynamical processes responsible for van der Waals interactions. However, their impact are usually small enough to justify the presented approach and we believe that they are not important in our particular case. As the issue of “strongly anharmonic mode” was previously observed and widely discussed on the example of the PbTe TO mode, we followed this lead and limited our study to that specific case. For comparison we included also longitudinal acoustic (LA) mode, which is far more harmonic (i.e. has potential which is closer to quadratic).

**Figure 1:**
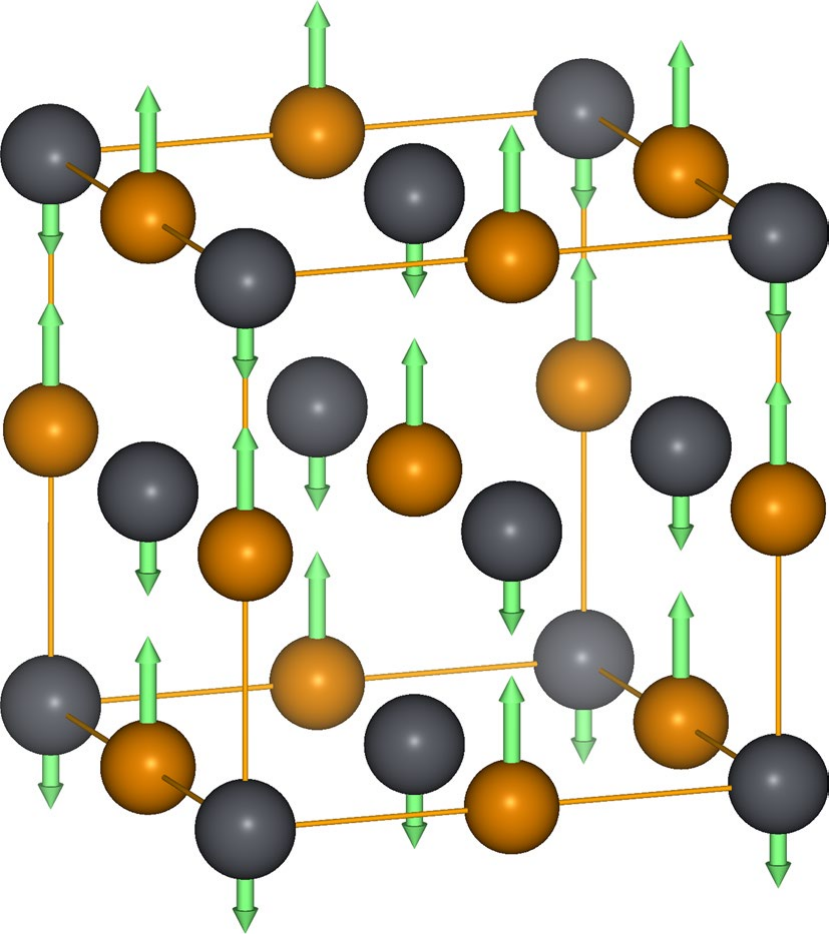
PbTe crystallographic (conventional) unit cell with TO mode polarization vector at the $\Gamma$ point (Brillouin zone center) visualized using green arrows. The gray and orange spheres depicted Pb and Te atoms, respectively.

Lead tellurate has exceptional thermoelectric properties (low thermal conductivity) suspected to be tied with anharmonic lattice dynamics, mainly with unusual behavior of the TO mode near the Brillouin zone center. The atomic displacements associated with that mode are schematically presented in Fig. 1. PbTe has a very simple rock salt crystal structure with high-symmetry cubic crystallographic unit cell which is easy for calculations. Additionally, a wide experimental database on its dynamical and thermodynamical properties, provides data for analysis and verification^11,13,33,34,40–42^.

Our paper is organized as follows: first we explain the calculation methods used in our work, next section contains main results with standard harmonic calculation in first subsection and detailed analysis of 1D and 2D mode potential in second subsection. We finish with discussion and conclusions in the last section.

## Calculation details

The basic property of a harmonic oscillator is the independence of its frequency from the amplitude of oscillations. Using so called direct method^29,43^ one can calculate phonon dispersion relations in harmonic approximation displacing all non-equivalent atoms in the structure in all symmetry independent directions, one at the time. Each displacement results in forces on all atoms in the system, which, in total, uniquely define force constants describing interaction in the crystal. Fourier transform of force constants defines dynamical matrix, which after diagonalization gives harmonic phonon frequencies as eigenvalues and polarization vectors as eigenvectors. If some normal mode of the crystal has a potential energy curve which is not strictly quadratic, the non-quadratic terms will show up as additional, non-linear terms in restoring forces for the mode and will lead to changes of the frequency with the amplitude of vibrations. Since direct method of phonon calculation is based on deriving frequencies of the normal modes directly from restoring forces, we can extract this information by calculating phonon dispersion relations for different amplitudes of displacement. Any changes in the mode frequency will indicate anharmonicity, or non-quadratic shape, of the mode potential.

For the ab initio calculation of the forces and energies we have used Density Functional Theory method as implemented in Vienna Ab Initio Simulation Package (VASP)^44,45^. The crystal structure was represented by a 2$\times$2$\times$2 supercell (constructed from the cubic crystallographic unit cell, depicted in Fig. 1) containing 64-atoms. The optimised lattice parameter, $a=6.557$ Å, matches well the experimental value of 6.46 Å. The exchange-correlation potential used PAW-PBE parametrization and atomic data sets^46–49^. The plane wave basis was limited to 300 eV energy cutoff. The reciprocal space integration was done over 4$\times$4$\times$4 k-point grid generated with Monkhorst-Pack scheme^50^. The energy convergence criterion for electronic structure calculation was set at $10^{-8}$ eV.

### Phonon frequencies versus amplitudes

**Figure 2:**
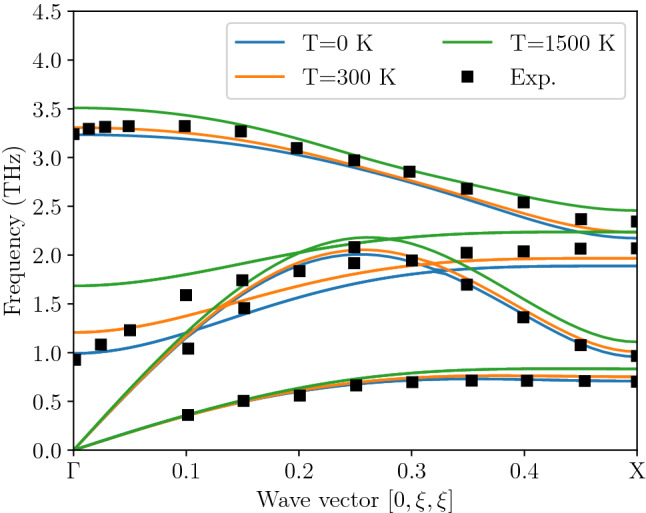
Phonon dispersion relations along $\Gamma$–X direction calculated with density functional theory and direct method^29,43^ using single-atom displacements equal to mean square displacements at 0, 300, and 1500 K (see Table 1). The points correspond to neutron measurements at 296 K^40^.

We started our study from standard phonon dispersion relations calculation^29,43^ based on single atom displacements equal to the self-consistent mean square displacements (see Table 1) corresponding to three different temperatures: 0, 300, and 1500 K.

**Table 1:** Self-consistently calculated mean square displacements for Pb and Te atoms in 0 K, 300 K, and 1500 K.

Temp. (K)	$\sqrt{\langle {u}_{\mathrm{Pb}}^{2} \rangle }$ (Å)	$\sqrt{\langle {u}_{\mathrm{Te}}^{2} \rangle }$ (Å)
0	0.043	0.043
300	0.149	0.126
1500	0.276	0.246

The results are presented in Fig. 2. The smallest displacement has been selected to be as close as possible to the small-vibrations regime, while keeping the force calculations accurate enough for mode frequency derivation. The other displacements are chosen to be rather large to enhance and clearly indicate any anharmonicity in the system. The dispersion curves in Fig. 2 show that any substantial anharmonicity connected with the shape of the potential energy surface seems to be mostly confined to the optical modes, particularly transverse optical (TO) mode. The acoustic modes stay almost constant over the whole range of amplitudes despite the fact that displacements corresponding to $T=1500$ K (c.f. Table 1) substantially deform the crystal. The above result is only a qualitative one—we cannot learn much about the shape of the potential and the scale of anharmonicity, we can just detect which branches move with changing displacement amplitude and so break harmonic approximation role. To learn more we need to investigate the energy surface of the mode in more detail. From this point on we will concentrate on the TO mode at the Brillouin zone center—as the apparent anharmonicity of this vibration is the largest (see Fig. 2) and where the claimed gigantic anharmonic effect^18^ is linked with this mode.

### TO mode potential

**Figure 3:**
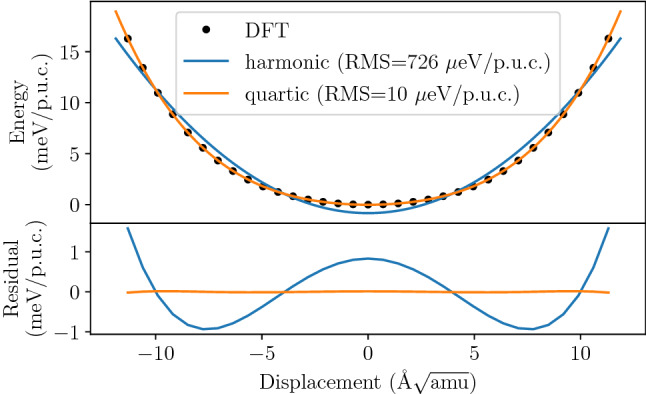
Potential of the TO mode at the $\Gamma$ point. The lines represent quadratic and quartic (fourth order) polynomial fits to the DFT data points. The residuals of the fits (bottom panel) indicate that the quartic fit is preferable.

To reconstruct the shape of mode potential for the TO mode at the $\Gamma$ point we have performed a series of DFT calculations for structures modulated by the TO mode and plotted the resulting energies, relative to the energy of the equilibrium configuration, as a function of mode amplitude (Fig. 3, top panel).

The residuals of the quadratic ($2 \mathrm{nd}$ order) and quartic ($4 {\mathrm{th}}$ order) polynomial fits to the DFT data (Fig. 3, top panel) clearly indicate that the simple quadratic (harmonic) function is not sufficient to properly reproduce the shape of the mode potential, while the fourth order polynomial provides a much better model of the DFT data points. The corresponding RMS errors of both fits are $726$ μeV/primitive unit cell (p.u.c.) and $10$ μeV/p.u.c., respectively.

In crystals the anharmonic component may take various forms. In many materials (e.g. $\hbox {ScF}_2$, $\hbox {TiO}_2$^51,52^) one or more of vibration modes is characterized by a strongly anharmonic potential of the general form of quartic oscillator potential for the normal mode coordinate *q*:

2 $$\begin{aligned} V(q)=\frac{\omega ^2}{2}q^2 + \frac{\lambda }{4}q^4. \end{aligned}$$

Our preliminary conclusion, based on the results of the phonon mode potential calculation above (Fig. 3), is that the one dimensional potential of the TO mode in PbTe resembles a fourth order polynomial of the quartic potential (Eq. 2) with sufficient accuracy. The results in Fig. 3 show substantial divergence of the energy surface from quadratic behaviour and good quality of quartic fit to the DFT data. The equation of motion for this potential can be solved analytically and the result can be further analyzed to obtain experimentally verifiable properties: mode frequency as a function of temperature, thermal displacements, line profile etc. The derivation of the analytic solution is provided in the auxiliary materials to this work. Unfortunately, the TO mode in PbTe at the zone center and along $\Gamma$–X direction is a doubly degenerate mode. Thus, the full mode potential is a function of two normal coordinates $V(q_1,q_2)$ and, in general case, cannot be separated into product or sum of two one-dimensional functions. The analytic solution may still be used for preliminary studies limited to one dimensional cuts of the energy surface (e.g. along the $q_1$ or $q_1 + q_2$ axis), but in cases, where the potential does not allow for its use, the procedure can still be carried out, however with higher computational cost, using numerical integration of the equation of motion.

**Figure 4:**
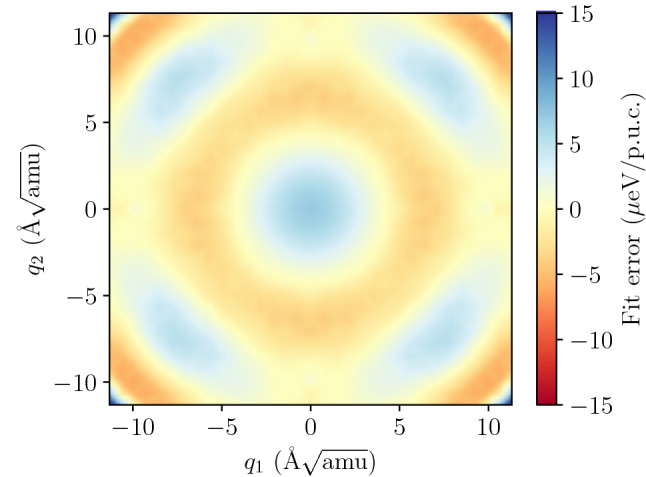
Residuals of the sixth order potential $V_6$ fit relative to the DFT energies of the TO mode at $\Gamma$ (in μeV/p.u.c.).

The numerical derivation of the solution requires careful definition of all the dimensional parameters in the formulas to provide physically meaningful results. Particularly, the normal coordinates ($q_1,q_2$) and potential parameters ($\omega ,\lambda$). In harmonic approximation the units of normal coordinates are less important in practice, since the vibration frequency does not depend on the amplitude of oscillations. In the anharmonic case the amplitude cannot be eliminated from the solution.

The coordinates $q_n$ of the normal mode *n* of harmonic crystal are related to the reduced Cartesian displacements $\sqrt{m_i}\,{\mathbf {u}}_i(t=0)$ of the *i*th atom in the primitive unit cell by the formula^1^:

$$\begin{aligned} \sqrt{m_i} u_i^\alpha = q_n e_{ni}^\alpha \; ; \quad q_n = \sum _{i\alpha }\sqrt{m_i} \, u_i^\alpha \, e_{ni}^\alpha \end{aligned}$$

where $e_{ni}^\alpha$ is an *i*th component of the *n*th normal mode polarization vector in direction $\alpha =x,y,z$; and $m_i$ is the mass of the *i*th atom. Note that in normal coordinates $q_n$ the mass of the oscillator is absorbed into the coordinates and thus final energy formula for quartic oscillator (Eq. 2) lacks the mass coefficient in the quadratic term. Note also that the unit of normal coordinate is $[\mathrm {length}\cdot \sqrt{\mathrm {mass}}]$ (e.g. Å$\sqrt{\mathrm {amu}}$ in our case).

The potential energy of the TO mode in the $Fm{\bar{3}}m$ crystal like PbTe could be expressed in terms of normal coordinates $q_1$, $q_2$. Furthermore, the symmetry constrains and Landau’s theory of phase transitions impose that the mode potential must be expressed in terms of two symmetry invariants:

$$\begin{aligned} i_3=q_1^2 + q_2^2 \; ; \; i_6=q_1^4 + q_2^4 \end{aligned}$$

Thus, general expansion of the potential to the *N*th order in $i_3, i_6$ reads:

3 $$\begin{aligned} V_N(q_1,q_2)=\sum _{l=0}^{\lfloor {\frac{N}{2}}\rfloor } i_3^{l}(q_1,q_2) \sum _{n=0}^{\lfloor {\frac{N}{4} - \frac{l}{2}}\rfloor } i_6^{n}(q_1,q_2) \, a_{l,n} \end{aligned}$$

We will limit the expansion to the sixth order:

4 $$\begin{aligned} V_{6} = i_{3}^{3} {a}_{3,0} + i_{3}^{2} {a}_{2,0} + i_{3} i_{6} {a}_{1,1} + i_{3} {a}_{1,0} + i_{6} {a}_{0,1} + {a}_{0,0} \end{aligned}$$

since the higher order terms did not provide any improvements in modeling of the calculated energy surface above numerical accuracy of the data points. Further analysis requires calculation of the energy surface of the TO mode at various amplitudes (i.e. values of the $q_i$ coordinates) and fitting of the $a_{l,n}$ coefficients of the model potential.

**Figure 5:**
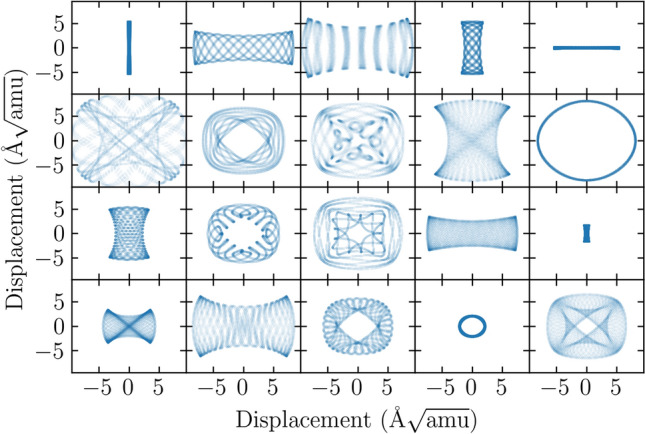
Sample of trajectories of the TO mode at the $\Gamma$ point, vibrating in the fitted $V_6$ potential (Eq. 4) at temperature $T=600$ K.

The calculation of the mode energy surface follows the same basic phonon scheme used above for the one dimensional case and involved calculation of the polarization vector for the mode and imposing the corresponding displacements of various amplitude onto the structure. The energy difference induced by the modulation of the structure has been calculated for a set of normal coordinates ($q_1,q_2$) on the regular 31$\times$31 grid for mode coordinates $|q_i| \le 11.3\,$Å$\sqrt{\mathrm {amu}}$ (approx. $3\sigma$ of the position distribution variance at $T=700$ K). The model potential (Eq. 4) was fitted to the data points using standard Levenberg-Marquardt curve-fitting implemented in SciPy library^53,54^. The RMS error of the fit was $3.02$ μeV/p.u.c.—which we consider to be a very good representation of the calculated data points (see Fig. 4).

The representation of the mode potential obtained in this way has been used to construct the equation of motion of the mode. This equation was integrated for given initial conditions using standard lsoda^55^ algorithm from the odepack^56^ library. The sample trajectories obtained with this procedure are presented in Fig. 5.

**Figure 6:**
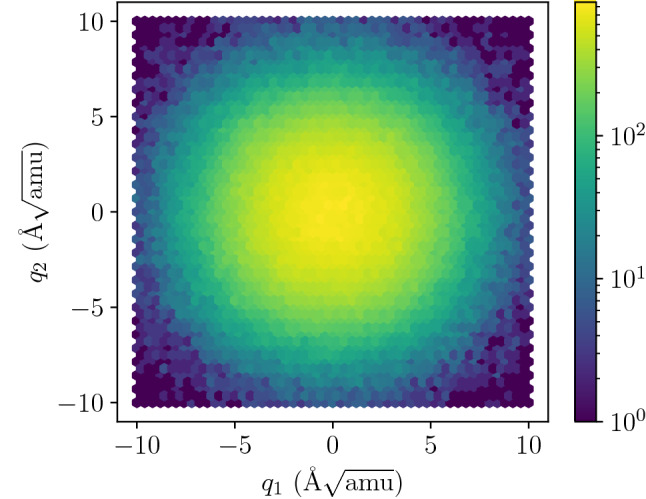
Mode coordinates density in the ensemble of 10,000 oscillators at $T=600$ K calculated from the trajectories of 30 ps length (8192 time steps). The oscillators correspond to TO mode at $\Gamma$ point. The color scale is logarithmic in probability density.

The trajectory of the system for one set of initial conditions is not enough to obtain such properties as spectral line shape of the mode for the system at given temperature. The real system contains many oscillators following different trajectories and interacting with each other. Our model does not include direct interactions of oscillators, as we have assumed that the interactions are small enough to justify the separate normal-modes approximation. The direct consequence of such assumption is that we obtain *intrinsic* mode parameters (e.g. line shape) stemming from the shape of the mode potential energy surface and energy distribution in the system. Thus, we model the crystal by the ensemble of oscillators with the kinetic energy following the Boltzmann distribution for given temperature. The simulation procedure involved generating the initial state ($q_1,q_2,{\dot{q}}_1,{\dot{q}}_2$) with positions and velocities distributed according to the normal distribution with variance adjusted to the value giving mean potential and kinetic energy equal to $k_B T/2$ per degree of freedom. The procedure was designed in this way to generate a set of configurations sampling thermal equilibrium state.

**Figure 7:**
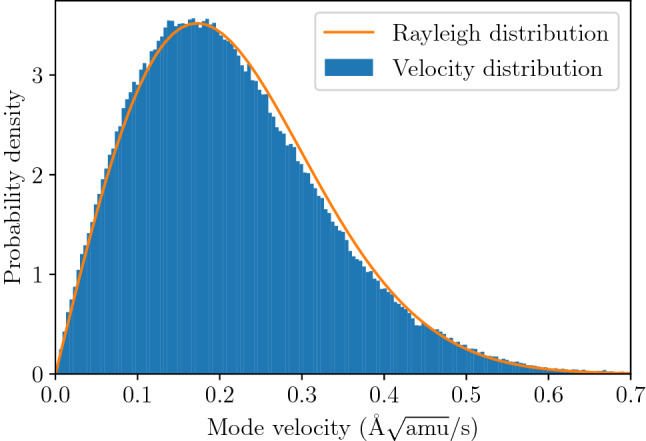
Velocity distribution for mode coordinates in the ensemble of 10,000 oscillators at $T=300$ K calculated from the trajectories of 30 ps length (8192 time steps). The oscillators correspond to TO mode at $\Gamma$ point.

## Results and discussion

**Figure 8:**
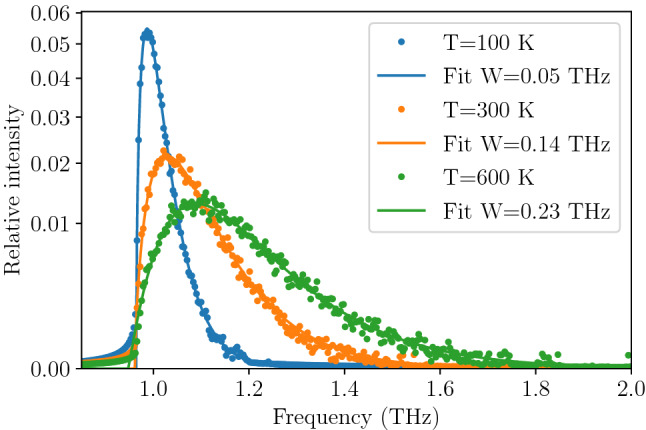
Intrinsic line profile of the TO mode in PbTe at $T=100{,}\, 300$, and $600\,$K calculated with an ensemble of 10,000 oscillators. The frequency resolution is determined by the length of the trajectory (30 ps). The typical experimental resolution is about 0.5 meV which corresponds to 0.12 THz (e.g. 0.3 meV reported by Manley et al.^58^ or 1.06 meV by Jensen et al.^8^). The vertical axis uses square scaling for better visibility of higher temperature peaks. The lines are fits of the asymmetrical line shapes to the data with full widths at half maximum (*W*) given in the legend.

We have used an ensemble of $10^4$ oscillators, each followed along 30 ps long trajectory (with time step $dt=3\,$fs). The final distribution of kinetic energy was measured to correspond to target temperatures of $T=100, 300,$ and $600\,$K with the accuracy of $\pm 15\,$K. The final probability density of position (Fig. 6) and velocity (Fig. 7) in the whole ensemble turned out to be adequately close to the target shapes—i.e. Gaussian distribution around equilibrium for position and Rayleigh (i.e. 2D Maxwell–Boltzmann) distribution for velocity—indicating that we have a good approximation of ensemble in thermodynamic equilibrium. For each trajectory the spectrum of velocity auto-correlation function has been calculated and averaged over the whole ensemble. The resulting spectral lines are presented in Fig. 8 together with fitted asymmetric line profiles^57^. The experimentally observed frequency of the TO mode is 0.93 THz^40^, which corresponds well with calculated energies in Fig. 8. Calculated profiles correctly mimic expected characteristic changes with temperature. The frequency of the mode and its FWHM significantly increases, while relative intensity decreases to conserve the area under profile. The rise of TO mode frequency corresponds well to predictions obtained from harmonic calculations presented in Fig. 2. Nevertheless, the calculated intrinsic line width of the TO mode, which stems from the variability of the mode frequency with oscillation amplitude, noticeably underestimates the experimental findings and can be compared with typical experimental resolution reported in literature. This result can be easily understood since presented profiles do not include any contribution originating from phonon-phonon interactions and represent exclusively pure effect of higher than quadratic terms in mode potential. It is worth noting that separation of such contributions is unavailable with any experimental technique and can be examined only with theoretical study.

On the other hand, one should keep in mind, that presented description of anharmonism is specific to the investigated system and neglects phonon-phonon interactions by design. The apparent inability to reconstruct so called “waterfall” effect in this approach indicates that these neglected interactions play significant role in creating this phenomenon and that it should not be ascribed to the anharmonicity of the TO mode itself.

The remaining possible sources of the broad and anomalous line shape of the TO mode (not covered in the DFT calculations presented above) include mainly changes in the local potential due to the normal mode interactions^7,22^, possible ferroelectric effects like micro-domains breaking local symmetry of the crystal and coupling to the TO mode^8,16,31,34,41^, multi-phonon interactions^21,32^, electron-phonon interactions^17^ or even phonon-strain coupling^15^. Finally we cannot also exclude possible dynamical effects not reproduced by the static (by definition) DFT calculation.

### LA mode profile

**Figure 9:**
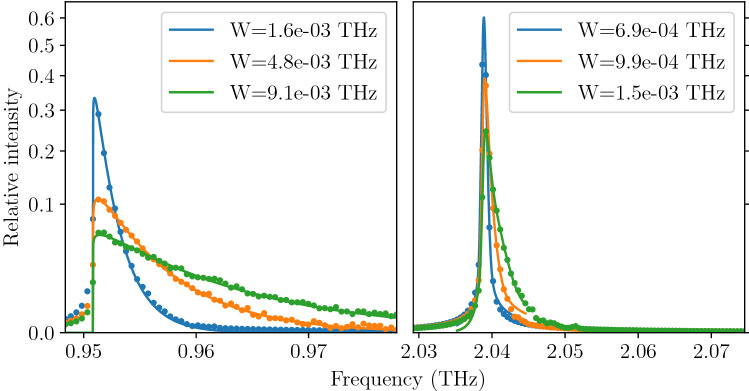
Intrinsic line profile of the LA mode in PbTe at $T=100, 300$, and $600\,$K calculated with an ensemble of 10,000 oscillators. The frequency resolution is determined by the length of the trajectory (200 ps). The line profile is calculated at two points in the Brillouin zone: X point at the zone boundary (left panel), middle of the zone in $\Gamma$–X direction (right panel). The dots mark the calculated spectra, the lines are fits of the asymmetrical line shapes to the data with full widths at half maximum (W) given in the legends. Note the difference in resolution of horizontal axis between above plot and the TO line plot. The vertical axis uses square scaling for better visibility of higher temperature peaks.

To validate our procedure in different case we have repeated above calculations for much more harmonic longitudinal-acoustic (LA) mode at two different points of the Brillouin zone. It is clearly visible from Fig. 2 that frequency changes generated by increasing atom displacement are one order of magnitude lower for LA than for TO mode. Also, fitting similar to that presented in Fig. 3 for TO mode leads to the small RMS error of the quadratic order (0.9 μeV/p.u.c.), while RMS error for quartic order potential is 0.01 μeV/p.u.c. which is close to the limit of the DFT energy convergence. The full spectral line calculations, presented in Fig. 9, further confirm expected high level of harmonicity of this mode, since the calculated intrinsic line widths are below 10 GHz.

The stark difference between profiles obtained for the TO (Fig. 8) and LA (Fig. 9) modes demonstrate the ability of the presented approach to extract the contribution from the mode anharmonicity to the shape of the phonon spectrum.

## Conclusions

The first-principle calculations presented above have demonstrated substantial dependence of TO phonon frequency on mode amplitude which is the marker of anharmonicity of the system. The shape of the mode potential at the center of the Brillouin zone has been mapped using single-point total energy calculations and fitted with sixth order polynomial function. Significant non-quadratic component of the obtained fit has confirmed anharmonic character of the phonon TO mode. In the next step an effective method to solve both 1D as well as 2D equation of motion and to derive phonon profile for the non-quadratic mode potential have been introduced. Obtained broadening of the TO phonon mode resulting from the change of the mode frequency with energy represents significant fraction of the experimental value, but does not reproduce observed TO line profile. This result does not support hypothesis that the anomalous line shape at the vicinity of the Brillouin zone center is caused by the anharmonicity of the singular mode potential^18^. On the other hand, the mode frequencies calculated, using the same DFT procedure, in the presence of all other modes show strong variations with the amplitude of the atomic displacement. This result points to the conclusion that in this case the basic assumption of the harmonic approximation—the independence of normal modes—breaks down. Furthermore, this phenomenon may manifest itself in dramatic and somewhat unexpected way, and is probably not limited to the case of PbTe crystal (e.g. PbSe^31^) or even to the family of similar compounds. This observation may be also used as additional validation of some form of ensemble sampling^27,31^ as a method of investigation of anharmonic effects using lattice dynamics. On the flip side, we see this result as an indicator that the methods based on single-atom or other non-physical displacements may be unable to capture essential parts of the lattice dynamics in such cases.

Presented arguments prompt us to the additional conclusion that, especially in the PbTe case, anharmonism of the system as a global feature should be rather associated with phonon-phonon interactions than with the particular mode and the shape of its potential.

## Supplementary information

Supplementary information
