# Supplementary material for: Phonon mode potential and its contribution to anharmonism
Source: Sci Rep. 2020 Nov 13;10:19783. doi: 10.1038/s41598-020-76454-y (PMC7666162; doi:10.1038/s41598-020-76454-y)
Supplement: Supplementary file 1 — Supplementary information [file 41598_2020_76454_MOESM1_ESM.pdf]

Phonon mode potential and its contribution to anharmonism

Paweł T. Jochym[1,*], Jan Łażewski[1], and Wojciech Szuszkiewicz[2,3]

[1]Institute of Nuclear Physics, Polish Academy of Sciences, Radzikowskiego 152, 31–342 Cracow, Poland
[*]pawel.jochym@ifj.edu.pl
[2]Institute of Physics, College of Natural Sciences, University of Rzeszów, Pigonia 1, 35–310 Rzeszów, Poland
[3]Institute of Physics, Polish Academy of Sciences, Lotników 32/46, 02–668 Warsaw, Poland

ABSTRACT

We present analytical solution of the harmonic-quartic oscillator equation of motion. While this is a standard mathematical result it is not easily available in the physics literature.

Quartic oscillator equation of motion

The anharmonic component of the interatomic potential may take various forms. In many materials (e.g. ScF_2, TiO_2) one or more of vibrational modes is characterized by a strongly anharmonic potential of the general form of quartic oscillator potential:

$$V(u) = \frac{m\omega^2}{2}u^2 + \frac{\lambda}{4}u^4, \tag{1}$$

which leads to the equation of motion for the oscillator of mass m and basic frequency ω, with anharmonic part scaled by λ':

$$m\ddot{u} + m\omega^2 u + \lambda' u^3 = 0. \tag{2}$$

This equation of motion can be solved analytically and the result can be further analyzed to obtain experimentally verifiable properties: mode frequency as a function of temperature, thermal displacements, line profile etc.

Even in cases where the potential does not allow for analytic solution, the procedure can still be carried out, however with higher computational cost, using numerical integration of the equation of motion. The analytic solution of the equation (2) has the form of the elliptic Jacobi function cn (for $\lambda > 0$ – which is a physically interesting case of the stable system):

$$u(t) = A\,\mathrm{cn}(t\Omega, \mu) \tag{3}$$

where:

$$\Omega = \sqrt{\omega^2 + \frac{\lambda}{m}A^2} \;;\; \mu = \sqrt{\frac{\lambda}{2m}\left(\frac{A}{\Omega}\right)^2} \tag{4}$$

This result comes from the properties of the $\mathrm{cn}(x,k)$ function which is a solution to the following differential equation:

$$\frac{d^2y}{dx^2} = (2-k^2)y - 2y^3,$$

which can be transformed into (2) using Ω, μ defined in (4). The period of oscillations is defined by the properties of the elliptic Jacobi functions cn. The general elliptic Jacobi function is a function of complex argument u and a parameter k known as an *elliptic modulus* ($0 < k^2 < 1$). The cn function is periodic, with periods $K(k)$ and $K'(k) \equiv K(\sqrt{1-k^2})$ in real and imaginary part, respectively:

$$\mathrm{cn}\left(u + 2\left(lK(k) + inK'(k)\right), k\right) = (-1)^{(l+n)}\mathrm{cn}(u,k)$$

where $K(k)$ is the complete elliptic integral of the first kind:

$$K(k) = \int_0^{\frac{\pi}{2}} \frac{1}{\sqrt{1-k^2\sin^2(\theta)}}\,d\theta$$

or in series form:

$$K(k) = \frac{\pi}{2}\sum_{n=0}^{\infty}\left(\frac{(2n-1)!!}{(2n)!!}\right)^2 k^{2n}.$$

Thus, if we limit our considerations to real arguments the period T and frequency ν of the cn function is:

$$T = 4K(\mu) = \frac{1}{\nu}$$

From the properties of the cn and K functions we can derive two cross-check limiting cases. A vanishing λ term (i.e. harmonic oscillator), and a small vibrations regime ($A \to 0$) oscillator with non-vanishing quartic term:

$$\lim_{\lambda\to 0}\nu = \lim_{A\to 0}\nu = \frac{\sqrt{\omega^2}}{2\pi},$$

Which are the expected value for harmonic oscillator. Note that the cn function becomes just standard cosine of the harmonic oscillator in these limiting cases.
